# Clinical safety and efficacy of simultaneous bilateral total knee arthroplasty in an Asian population: a propensity score-matched analysis

**DOI:** 10.1186/s13018-025-05933-7

**Published:** 2025-05-24

**Authors:** Kwan Kyu Park, Hyuck Min Kwon, Byung Woo Cho, Tae Sung Lee, Woo-Suk Lee, Jun Young Park

**Affiliations:** 1 Department of Orthopaedic Surgery, Severance Hospital, Yonsei University College of Medicine, Seoul, Republic of Korea; 2 Department of Orthopaedic Surgery, Gangnam Severance Hospital, Yonsei University College of Medicine, Seoul, Republic of Korea; 3 Department of Orthopaedic Surgery, Heung-K Hospital, Gyeonggi-do, Republic of Korea; 4 Department of Orthopaedic Surgery, Yongin Severance Hospital, Yonsei University College of Medicine, 363, Dongbaekjukjeon-daero, Giheung-gu, Yongin-si, 16995 Gyeonggi-do Republic of Korea

**Keywords:** Total knee arthroplasty, Simultaneous surgery, Knee osteoarthritis, Postoperative complication, Blood transfusion, Mortality

## Abstract

**Background:**

Clear clinical guidelines on performing simultaneous bilateral total knee arthroplasty (BTKA) are lacking. We compare the clinical outcomes between BTKA and unilateral total knee arthroplasty (UTKA) using propensity score matching to assess safety and clinical efficacy, hypothesizing no difference in clinical safety.

**Methods:**

Among 1,665 BTKA and UTKA cases, patients were matched in a 1:1 ratio by age, sex, body mass index, follow-up, and comorbidities, resulting in 653 patients per group. Primary outcomes included 30-day complication rates and intensive care unit (ICU) admission rates. Secondary outcomes included length of stay (LOS), transfusion rate, estimated blood loss, hemoglobin (Hb) levels (preoperative and two days postoperative), Hb decrease, and 1-year mortality rate. The patient-reported outcomes (PROMs) was measured preoperatively and at 3, 6, and 12 months postoperatively using the American Knee Society Score, Western Ontario and McMaster Universities Osteoarthritis Index, and EuroQol 5-Dimension.

**Results:**

There were no differences in the 30-day complication rates and ICU admission rate between the BTKA and UTKA groups after matching (1.4% vs. 0.9%; *p* = 0.60, 0.5% vs. 0.6%; *p* = 1.00). However, patients who underwent BTKA had a longer LOS, a higher incidence of transfusion (7.2% vs. 2.1%; *p* < 0.001), greater blood loss (128.6 ± 75.5 vs. 72.5 ± 45.6 mL; *p* < 0.001), and a more pronounced decrease in Hb levels (3.1 vs. 2.9 g/dL; *p* < 0.001) than those who underwent UTKA. No significant differences were observed in PROMs at one year postoperatively.

**Conclusions:**

Patients who underwent BTKA reported similar 30-day complication rates, ICU admissions, and PROMs compared to UTKA. Despite higher LOS, transfusion rates, blood loss, and Hb decrease, BTKA remains a safe, effective option. It should be performed cautiously, considering patient comorbidities and overall health in treating bilateral knee OA.

**Supplementary Information:**

The online version contains supplementary material available at 10.1186/s13018-025-05933-7.

## Background

Total knee arthroplasty (TKA) is a widely performed, cost-effective procedure for severe knee osteoarthritis (OA), with its utilization steadily increasing due to advances in surgical techniques, population aging, and rising activity demands [1–3]. Unilateral OA may accelerate contralateral knee joint disease through increased joint loading with the hypercontraction of thigh muscle and abnormal gait pattern [4]. Knee OA frequently affects both knees over time, even if it begins as a unilateral condition [5]. In particular, a 12-year follow-up study found that approximately 70% of participants with chronic knee pain developed bilateral radiographic OA, with 80% of those initially diagnosed with unilateral OA progressing to bilateral disease during follow-up [6]. Additionally, contralateral knee joint OA was observed in about 90% of TKA and unicompartmental knee arthroplasty cases [7, 8].

Bilateral TKA for severely dysfunctional bilateral knee joints can be conducted as simultaneous, staggered, or staged procedures [9]. Simultaneous bilateral TKA (BTKA), which involves conducting both procedures under a single anesthetic session, provides benefits such as improved cost efficiency, a unified rehabilitation period, and reduced overall hospital stay [10–13]. However, studies show mixed results regarding BTKA safety. Some report comparable complications and functional outcomes to staged procedures, while others document higher rates of morbidity and mortality [14–16]. Studies based on the National Inpatient Sample database reported that the usage of BTKA more than doubled since the 2000s among three surgical options for bilateral TKA; however, the frequency of BTKA in the 2010s was approximately 4–5% of all cases [17]. Additionally, some experts have suggested that a systematic approach should be implemented when deciding on BTKA to reduce patient complications due to increased medical risks [18].

Therefore, clear clinical guidelines have not yet been established on whether to perform bilateral TKA simultaneously or in a staged procedure [19]. As far as we know, there is a lack of evidence regarding the clinical safety and efficacy of BTKA compared to unilateral TKA (UTKA) in the Asian population.

We sought to elucidate the clinical efficacy of BTKA by comparing clinical parameters such as the 30-day complication rate, intensive care unit (ICU) admission rate, length of stay (LOS), transfusion rate, degree of hemoglobin (Hb) decrease and 1-year mortality between the BTKA and the UTKA groups. This comparison was conducted using propensity score matching to control for potential biases, including age, sex, and body mass index (BMI). We hypothesize that there will be no difference in clinical outcomes related to safety between the BTKA and UTKA groups.

## Methods

### Study patient selection

This retrospective case-control study compared the clinical parameters between the BTKA and UTKA groups. This study was approved by the Institutional Review Board of Severance Hospital, Seoul, South Korea (2024). We searched the electronic database of our institution to identify all patients who had undergone BTKA or UTKA between January 2018 and December 2022. Eligible patients had an OA or spontaneous osteonecrosis of the knee diagnosed via radiographic examination, were aged over 50, and decided to undergo TKA due to unrelieved pain and significant functional loss. Exclusion criteria included severe joint-destructive diseases such as rheumatoid arthritis and hemophilic arthritis, a history of knee joint infection, periarticular trauma requiring surgery, and an inability to follow up for more than one year. After selection, a total of 1,665 cases were enrolled, including 659 BTKA cases (39.6%) and 996 UTKA cases (59.8%). The primary indication for TKA in this cohort was end-stage knee OA. Specifically, among the UTKA group, 8 patients (0.8%) had osteonecrosis as the primary diagnosis. No cases of osteonecrosis were identified in the BTKA group.

### Data collection

We defined major complications as surgical complications requiring revision surgery or resulting in death and medical complications with potential mortality occurring within 30 days postoperatively. Minor complications included surgical issues not requiring revision surgery, medical conditions manageable with conservative treatment, and delayed discharge exceeding twice the expected LOS. Complication rates were measured by combining the number of major and minor complications. We also collected data on the ICU admission rate during the postoperative hospitalization period, LOS, transfusion rate, estimated blood loss, preoperative and postoperative Hb levels, the degree of Hb decrease up to postoperative day two, and 1-year mortality. Demographic parameters collected included age, sex, BMI, follow-up period, use of suction drain, and the American Society of Anesthesiologists (ASA) classification, which assesses patients’ physical health on a scale from 1 to 4, with 1 indicating the healthiest status. At study entry, baseline American Knee Society Score (AKSS), Western Ontario and McMaster Universities Osteoarthritis Index (WOMAC), and EuroQol 5-Dimension (EQ5D) scores were obtained [20]. AKSS, WOMAC, and EQ5D scores were also obtained at preoperative, 3, 6, and 12 months postoperatively.

### Decision of type of TKA and perioperative management

The type of surgery, either BTKA or UTKA, was determined based on the osteoarthritis status of both knees and the patient’s preference. In the absence of hospital guidelines for BTKA eligibility, factors such as comorbidities and age were not considered when deciding between BTKA and UTKA. For patients requiring bilateral surgery, most procedures were performed simultaneously. However, simultaneous surgery was not conducted if patients declined it during preoperative counseling. All patients underwent a standard preoperative evaluation of comorbidities and ASA classification conducted by a consultant anesthesiologist and internist several weeks before surgery. For patients who have high risk, we consulted relevant internists and conducted additional assessments, including echocardiography, pulmonary function tests, and further laboratory blood tests. Based on these findings, appropriate internal medical treatments were applied. Postoperatively, discharge was approved once the patient could ambulate independently with a walker and manage pain with oral medication.

### TKA procedure

The TKA procedures were performed by two high-volume surgeons, both of whom were fellowship-trained in hip and knee arthroplasty, shared surgical techniques, and worked with the same surgical team. All surgeries utilized a midline anterior incision with a medial parapatellar approach. Pneumatic tourniquets and suction drainage were applied in every case, and patellar resurfacing was not performed. In cases of BTKA, the tourniquet on the second leg was inflated after the first tourniquet was released. Intravenous tranexamic acid was administered during the perioperative phase to reduce blood loss and the need for transfusion. For alignment, an intramedullary system was used for the femur, and an extramedullary system was used for the tibia. Periarticular multimodal drug injections and peripheral nerve blocks were administered to manage postoperative pain, following the enhanced recovery after surgery protocol of our arthroplasty department [21, 22].

### Study endpoints

The primary outcomes were the complication rates within the first 30 days postoperatively and the ICU admission rate. The secondary outcomes included LOS, transfusion rate, estimated blood loss, preoperative and postoperative Hb levels on postoperative day two, the degree of Hb decrease from preoperative baseline to postoperative day two, 1-year mortality, and PROM preoperatively and at 3, 6, and 12 months postoperatively.

### Statistical analyses

We used the *t-test* and Mann-Whitney U test for continuous variables as well as the *chi*-square test and Fisher’s exact test for categorical variables to compare the mean and proportion of selected baseline characteristics. A propensity score matching analysis was conducted to minimize biases, using single nearest-neighbor matching where each unilateral TKA case was matched to a bilateral TKA participant with the closest baseline characteristics [23]. Patients who underwent BTKA were matched to those who underwent UTKA in a 1:1 ratio based on age, sex, BMI, follow-up periods, and ASA. After matching, 653 BTKA and 653 UTKA cases were identified. Statistical significance was defined as *P* < 0.05. All statistical analyses were performed using R software, version 4.4.0.

## Results

### Cohort characteristics after matching

After matching, 1,306 patients were included, with 653 patients in the BTKA group and 653 in the UTKA group (Table 1). The mean age and BMI (± standard deviation) were 71.8 ± 5.4 years and 26.7 ± 2.4 kg/m² for the BTKA group and 71.6 ± 5.9 years and 26.6 ± 2.2 kg/m² for the UTKA group (*p* = 0.61 and *p* = 0.45, respectively). The proportion of female patients was 86.4% in the BTKA group and 86.2% in the UTKA group (*p* = 1.0).

**Table 1:** Demographics of the study cohort

Variables	Before propensity score matching				After propensity score matching			
	Simultaneous bilateral TKA group	Unilateral TKA group	Total	*P*		Simultaneous bilateral TKA group	Unilateral TKA group	*P*
Numbers of knees	659	996	1655			653	653	
Age (years)	71.7 ± 5.5	73.3 ± 6.5	72.6 ± 6.2	< 0.01		71.8 ± 5.4	71.6 ± 5.9	0.61
Female, n (%)	570 (86.5)	812 (81.5)	1382 (83.5)	0.01		564 (86.4)	563 (86.2)	1.00
BMI (kg/m^2^)	26.8 ± 2.5	26.5 ± 2.1	26.6 ± 2.3	0.02		26.7 ± 2.4	26.6 ± 2.2	0.45
ASA classification, n (%)				0.68				0.45
1	12 (1.8)	21 (2.1)	33 (2.0)			12 (1.8)	19 (2.9)	
2	316 (48.0)	475 (47.7)	791 (47.8)			313 (47.9)	308 (47.2)	
3	331 (50.2)	498 (50.0)	829 (50.1)			328 (50.2)	325 (49.8)	
4	0 (0.0)	2 (0.2)	2 (0.1)			0 (0.0)	1 (0.2)	
Follow up period, years	4.2 ± 1.5	4.1 ± 1.4	4.1 ± 1.4	0.3		4.2 ± 1.5	4.1 ± 1.4	0.39
Drain use, n (%)	650 (98.6)	978 (98.2)	1628 (98.4)	0.62		644 (98.6)	640 (98.0)	0.52

TKA, total knee arthroplasty; BMI, body mass index; ASA, American Society of Anesthesiologists

### Complications and ICU admission rate related to BTKA

After matching, the BTKA cohort showed a similar rate of complications within the first 30 days postoperatively compared to the UTKA cohort (1.4% vs. 0.9%; *p* = 0.60) (Table 2). Before matching, the complication rates between the two groups were also comparable (1.5% vs. 1.2%; *p* = 0.75) (see Supplementary Table 1). Major complications occurred in 5 patients (0.8%) in the BTKA group, including periprosthetic joint infection (1 case), aseptic loosening (2 cases), pulmonary embolism (1 case), and hypovolemic shock (1 case), while in the UTKA group, there were 2 cases (0.3%), including pneumonia (1 case) (Table 3). Minor complications were observed in 4 patients (0.6%) within the BTKA group, including cases of wound dehiscence (1 case) and delayed discharge (3 cases). In the UTKA group, minor complications occurred in 4 patients (0.6%), including superficial infections (1 cases), prolonged wound drainage (1 case), and hemarthrosis (1 cases). There were no significant differences in ICU admission rates between the two groups before and after matching (0.5% vs. 0.5%; *p* = 1.00, 0.5% vs. 0.6%; *p* = 1.00).

**Table 2:** Comparison of clinical outcome parameters between simultaneous bilateral and unilateral total knee arthroplasty groups after propensity score matching

Variables	After propensity score matching			
	Simultaneous bilateral TKA group	Unilateral TKA group	*P*	
Numbers of knees	653	653		
30-day complication, n (%)	9 (1.4)	6 (0.9)	0.60	
ICU admission, n (%)	3 (0.5)	4 (0.6)	1.00	
Length of stay, days	3.9 ± 1.4	3.7 ± 1.1	< 0.01	
Transfusion required, n (%)	47 (7.2)	14 (2.1)	< 0.001	
Estimated blood loss, ml	128.6 ± 75.5	72.5 ± 45.6	< 0.001	
Hemoglobin				
Preoperative	12.5 ± 0.7	12.5 ± 0.5	0.34	
Postoperative day 1	10.3 ± 1.2	11.0 ± 1.2	< 0.001	
Postoperative day 2	9.4 ± 0.5	9.6 ± 0.5	< 0.001	
Hemoglobin decrease	3.1 ± 0.8	2.9 ± 0.7	< 0.001	
Mortality, n (%)	8 (1.2)	4 (0.6)	0.38	

TKA, total knee arthroplasty; ICU, intensive care unit

**Table 3:** Comprehensive summary of major and minor complications in simultaneous bilateral and unilateral total knee arthroplasty groups after propensity score matching

	Simultaneous bilateral TKA group	Unilateral TKA group	*p*
30-day Complication, n (%)	9 (1.4)	6 (0.9)	0.73
Major	5 (0.8)	2 (0.3)	0.45
Periprosthetic joint infection	1	1	
Aseptic loosening	2	0	
Pulmonary embolus	1	0	
Hypovolemic shock	1	0	
Pneumonia	0	1	
Minor	4 (0.6)	4 (0.6)	1.00
Superficial infection	0	1	
Wound dehiscence	1	0	
Prolonged wound drainage	0	1	
Hemarthrosis	0	1	
Delayed discharge	3	1	

TKA, total knee arthroplasty

### Comparison of secondary outcomes between the BTKA and UTKA groups

After matching, patients in the BTKA group exhibited a longer LOS (3.9 ± 1.4 days vs. 3.7 ± 1.1 days; *p* < 0.01), a higher transfusion rate (7.2% vs. 2.1%; *p* < 0.001), greater estimated blood loss (128.6 ± 75.5 ml vs. 72.5 ± 45.6 ml; *p* < 0.001), and a more significant decrease in Hb levels (3.1 ± 0.8 g/dL vs. 2.9 ± 0.7 g/dL; *p* < 0.001) compared to the UTKA group (Table 2). However, no significant difference was found in the 1-year mortality rate between the two groups (1.2% vs. 0.6%; *p* = 0.384).

### Comparison of PROMs

There were no significant differences in the PROMs parameters at postoperative one year, such as AKSS knee (90.7 ± 5.2 vs. 90.4 ± 4.9; *p* = 0.214), AKSS function (78.9 ± 7.2 vs. 78.7 ± 5.7; *p* = 0.561), WOMAC (19.1 ± 7.5 vs. 19.6 ± 7.1; *p* = 0.305), and EQ5D score (77.0 ± 7.4 vs. 77.1 ± 6.4; *p* = 0.731) (Table 4).

**Table 4:** Comparison of patient-reported outcome measures between two groups after propensity score matching

Variables	After propensity score matching			
	Simultaneous bilateral TKA group	Unilateral TKA group	*P*	
AKS knee score				
Preoperative	45.9 ± 9.5	45.7 ± 9.4	0.78	
Change at 3 m	38.7 ± 12.8	39.4 ± 11.7	0.27	
Change at 6 m	43.8 ± 11.8	43.7 ± 10.9	0.83	
Change at 1 year	44.8 ± 10.7	44.6 ± 10.2	0.72	
AKS function score				
Preoperative	40.5 ± 10.7	41.2 ± 9.1	0.18	
Change at 3 m	35.4 ± 13.7	34.5 ± 12.8	0.19	
Change at 6 m	37.0 ± 13.0	36.2 ± 11.3	0.27	
Change at 1 year	38.5 ± 13.3	37.5 ± 9.6	0.14	
WOMAC				
Preoperative	67.4 ± 12.8	66.5 ± 11.1	0.17	
Change at 3 m	-42.9 ± 15.7	-41.4 ± 14.4	0.07	
Change at 6 m	-44.0 ± 15.6	-43.8 ± 13.6	0.79	
Postoperative 1 year	-48.2 ± 14.1	-46.9 ± 13.1	0.08	
EQ5D				
Preoperative	45.2 ± 13.7	44.4 ± 12.1	0.27	
Change at 3 m	26.5 ± 15.8	27.0 ± 12.9	0.53	
Change at 6 m	30.3 ± 16.1	30.6 ± 13.8	0.76	
Change at 1 year	31.8 ± 15.9	32.7 ± 14.4	0.27	

TKA, total knee arthroplasty; AKS, American Knee Society; WOMAC, Western Ontario and McMaster Universities Osteoarthritis Index; EQ5D, EuroQol 5-Dimension

Changes are calculated as the difference between postoperative scores and baseline (preoperative) values. WOMAC is reported in a decreasing direction (lower scores indicate better outcomes)

## Discussion

Our study investigated the safety and clinical efficacy of BTKA in the Asian population compared with UTKA using propensity score matching. There were no differences in the complication rates during the first postoperative 30 days and ICU admission rates between the BTKA and UTKA groups after matching. However, patients who underwent BTKA had a longer LOS, a higher incidence of transfusion, greater blood loss, and a more pronounced decrease in Hb levels than those who underwent UTKA. There were no significant differences in clinical outcomes at one year postoperatively, as measured by PROMs, including AKSS, WOMAC, and EQ5D scores.

Overall, patients who underwent BTKA reported similar short-term postoperative complication rates and functional gains compared to those who underwent UTKA. Although our sample size was substantial, it is important to note that the absence of statistically significant differences in these outcomes could potentially be influenced by limited statistical power, and Type II errors cannot be ruled out. Of note, our pre-matching analysis revealed that BTKA patients were generally younger, more often female, and had a slightly higher BMI compared to UTKA patients, highlighting the importance of propensity matching in our approach to minimize selection bias.

Recent meta-analyses have reported that BTKA is associated with higher odds of postoperative pulmonary embolism [12, 24], including one involving 18 articles and another utilizing 29 studies. Additionally, increased risks of thromboembolic events, myocardial infarction, and stroke have been noted in patients undergoing BTKA [25–27]. This study observed pulmonary embolism events within 30 days postoperatively in both the BTKA and UTKA groups. However, our study did not observe significantly higher rates of venous thromboembolism (VTE) in the BTKA group. Several unique characteristics of our cohort and protocol likely contributed to this finding. All surgeries were performed in a high-volume tertiary center with standardized fast-track protocols, including early mobilization and routine chemical thromboprophylaxis. Additionally, we employed consistent use of tranexamic acid, pneumatic tourniquets, and experienced surgical teams, which effectively controlled blood loss, a known risk factor for thromboembolic events. These factors may have mitigated the typical increased VTE risk associated with BTKA reported in broader populations. This aligns with findings from recent large-scale registry studies by Memtsoudis et al., which emphasize the importance of institutional protocols in lowering VTE incidence, even in high-risk procedures such as BTKA [28].

Although severe respiratory failure was not observed in our study, it has been reported as a serious complication following BTKA in other studies [29]. The occurrence of severe ischemic events may be influenced by differences in surgical approach, including increased surgical burden during a single anesthetic session, prolonged exposure to anesthesia, and higher ASA classification or Charlson Comorbidity Index in surgical patients [30].

BTKA has been associated with significantly higher intraoperative blood loss and transfusion rates compared to UTKA or staged bilateral TKA procedures, with some studies reporting transfusion volumes up to four times greater [14, 31, 32]. This study reported a higher risk of developing blood loss anemia and about three times more frequency of transfusions in patients who underwent BTKA, even with the perioperative use of tranexamic acid and tourniquets. This finding is likely attributable to the additive blood loss from two surgical sites and prolonged operative time under a single anesthetic exposure, which increases cumulative intraoperative bleeding. However, this study found no significant increase in blood loss-related major complications in BTKA, with an overall transfusion rate of approximately 7.2%, which was relatively low. Our transfusion findings are comparable to those reported by Abdel et al., which showed similar trends when modern hemostatic strategies such as tranexamic acid were employed [33].

Staged bilateral TKA was associated with a longer overall hospital stay than BTKA due to the interval between two surgeries [12]. An increase in LOS may ultimately be associated with a higher risk of postoperative complications. In this study, the LOS for BTKA was statistically significantly longer than the unilateral procedure, though the difference was not substantial. This slight increase in LOS may be attributed to the increased physical demands of bilateral rehabilitation, greater postoperative monitoring needs, and delayed functional recovery following more extensive surgical trauma. For our study participants, discharge was planned once a certain level of rehabilitation and pain control was achieved, regardless of the type of surgery, which may have minimized differences in LOS. Implementing our ERAS protocol, which includes preoperative patient optimization, opioid-sparing multimodal anesthesia, and early mobilization, likely contributed to the reduction in the LOS. The existing literature suggests that patients undergoing BTKA achieve comparable or even superior functional outcomes, including a range of motion and PROMs such as the KSS and WOMAC, compared to those undergoing staged bilateral TKA or UTKA [34, 35]. Our findings also demonstrated that BTKA yielded outcomes comparable to UTKA at 3, 6, and 12 months postoperatively.

The potential increase in postoperative mortality associated with BTKA remains a significant and contentious issue. Several studies have demonstrated that BTKA shows no significant differences in mortality compared to UTKA or staged bilateral TKA [36, 37]. This study showed no differences in 1-year mortality rates between the two groups before and after matching. In contrast, recent studies, including meta-analyses, have reported up to a threefold increase in mortality associated with BTKA [12, 38]. These differences may be attributed to variability in the cohort composition, such as individual baseline comorbidities (e.g., ASA classification), as well as differences in follow-up durations, such as 30 or 90 days.

Our study has several inherent limitations. First, although propensity score matching was employed to reduce selection bias, as a retrospective study, it was not possible to completely control for all biases between the two cohorts. A fundamental limitation stems from the inherent difference in disease laterality: patients undergoing BTKA had bilateral severe OA, whereas those undergoing UTKA likely had unilateral severe OA, with or without contralateral involvement. Consequently, BTKA patients may have had greater comorbidity burdens, older age, or other characteristics not fully accounted for by the available covariates. While propensity score matching helped mitigate these differences, residual and unmeasured confounding factors may still exist. Nonetheless, by consecutively collecting and analyzing patients during the study period and deciding on simultaneous surgery based on patient needs rather than strict guidelines, we minimized selection bias.​ Second, due to the limitations of a retrospective chart review, our analysis of postoperative complications was confined to clearly defined in-hospital complications occurring within the short-term period of 30 days post-surgery. We did not report on the incidence of complications in a long-term, prospective manner. Third, as our analysis did not compare staged bilateral TKA, we were unable to provide recommendations on the optimal duration between procedures when a staged approach is necessary. Prospective studies are needed to determine the optimal duration between the two surgeries in staged bilateral TKA. Finally, postoperative complications were primarily assessed in the short term, limiting our ability to observe longer-term outcomes such as sepsis, 90-day readmission, stroke, and myocardial infarction. Additionally, for all outcomes where no significant differences were observed between groups, including complications, mortality, and PROMs, the possibility of Type II error due to limited statistical power cannot be excluded, despite our relatively large sample size. Future studies with larger cohorts may be better positioned to detect subtle differences between these groups if they exist.

## Conclusions

In conclusion, patients who underwent BTKA reported similar postoperative 30-day complication rates, ICU admission rates, and comparable clinical satisfaction based on PROMs compared to UTKA. Despite the drawbacks of a longer LOS, higher transfusion rates, greater blood loss, and a marked decrease in Hb levels, BTKA can be considered effective and safe compared to UTKA. Thus, this procedure should be performed carefully, taking into account the patient’s comorbidities and overall physical condition when treating patients who have knee OA.

## Electronic supplementary material

Below is the link to the electronic supplementary material.

Supplementary Material 1

Supplementary Material 2

## Data Availability

No datasets were generated or analysed during the current study.
