# Supplementary material for: Clinical safety and efficacy of simultaneous bilateral total knee arthroplasty in an Asian population: a propensity score-matched analysis
Source: J Orthop Surg Res. 2025 May 24;20:508. doi: 10.1186/s13018-025-05933-7 (PMC12102920; doi:10.1186/s13018-025-05933-7)
Supplement: Supplementary file 1 — Supplementary Material 1 [file 13018_2025_5933_MOESM1_ESM.docx]

**Supplementary table 1.** Comparison of clinical outcome parameters between simultaneous bilateral and unilateral total knee arthroplasty groups before propensity score matching

| **Variables** | **Before propensity score matching** | | | | |
| --- | --- | --- | --- | --- | --- |
|  | **Simultaneous bilateral TKA group** | **Unilateral TKA group** | **Total** | ***P*** |  |
| **Numbers of knees** | 659 | 996 | 1655 |  |  |
| **Overall complication, n (%)** | 10 (1.5) | 12 (1.2) | 22 (1.3) | 0.75 |  |
| **ICU admission, n (%)** | 3 (0.5) | 5 (0.5) | 8 (0.5) | 1.00 |  |
| **Length of stay, days** | 3.9 ± 1.4 | 3.7 ± 1.2 | 3.8 ± 1.3 | <0.01 |  |
| **Transfusion required, n (%)** | 47 (7.1) | 24 (2.4) | 71 (4.3) | < 0.001 |  |
| **Estimated blood loss, ml** | 128.5 ± 75.4 | 72.0 ± 44.2 | 94.5 ± 64.8 | < 0.001 |  |
| **Hemoglobin** |  |  |  |  |  |
| **Preoperative** | 12.5 ± 0.7 | 12.5 ± 0.5 | 12.5 ± 0.6 | 0.13 |  |
| **Postoperative day 1** | 10.3 ± 1.2 | 10.9 ± 1.3 | 10.7 ± 1.3 | < 0.001 |  |
| **Postoperative day 2** | 9.4 ± 0.5 | 9.5 ± 0.5 | 9.5 ± 0.5 | < 0.001 |  |
| **Hemoglobin decrease** | 3.1 ± 0.8 | 2.9 ± 0.7 | 3.0 ± 0.7 | < 0.001 |  |
| **Mortality, n (%)** | 8 (1.2) | 8 (0.8) | 16 (1.0) | 0.56 |  |

TKA, total knee arthroplasty; ICU, intensive care unit
