# Supplementary material for: Clinical safety and efficacy of simultaneous bilateral total knee arthroplasty in an Asian population: a propensity score-matched analysis
Source: J Orthop Surg Res. 2025 May 24;20:508. doi: 10.1186/s13018-025-05933-7 (PMC12102920; doi:10.1186/s13018-025-05933-7)
Supplement: Supplementary file 2 — Supplementary Material 2 [file 13018_2025_5933_MOESM2_ESM.docx]

**Supplementary table 2.** Comparison of patient-reported outcome measures between two groups before propensity score matching

| **Variables** | **Before propensity score matching** | | | | |
| --- | --- | --- | --- | --- | --- |
|  | **Simultaneous bilateral TKA group** | **Unilateral TKA group** | **Total** | ***P*** |  |
| **AKS knee score** |  |  |  |  |  |
| **Preoperative** | 45.9 ± 9.5 | 45.8 ± 9.2 | 45.8 ± 9.3 | 0.77 |  |
| **Change at 3 m** | 38.7 ± 12.8 | 39.3 ± 11.3 | 39.1 ± 11.9 | 0.31 |  |
| **Change at 6 m** | 43.8 ± 11.8 | 43.7 ± 10.5 | 43.7 ± 11.0 | 0.77 |  |
| **Change at 1 year** | 44.8 ± 10.7 | 44.6 ± 10.1 | 44.7 ± 10.3 | 0.62 |  |
| **AKS function score** |  |  |  |  |  |
| **Preoperative** | 40.6 ± 10.7 | 41.3 ± 9.3 | 41.0 ± 9.9 | 0.16 |  |
| **Change at 3 m** | 35.3 ± 13.8 | 34.9 ± 12.5 | 35.1 ± 13.0 | 0.51 |  |
| **Change at 6 m** | 36.8 ± 13.2 | 36.2 ± 11.2 | 36.4 ± 12.0 | 0.30 |  |
| **Change at 1 year** | 38.4 ± 13.3 | 37.3 ± 10.2 | 37.7 ± 11.5 | 0.09 |  |
| **WOMAC** |  |  |  |  |  |
| **Preoperative** | 67.4 ± 12.8 | 66.5 ± 10.8 | 66.9 ± 11.7 | 0.17 |  |
| **Change at 3 m** | -42.9 ± 15.7 | -41.6 ± 14.2 | -42.1± 14.8 | 0.08 |  |
| **Change at 6 m** | -44.0 ± 15.6 | -43.7 ± 13.5 | -43.8 ± 14.4 | 0.70 |  |
| **Postoperative 1 year** | -48.2 ± 14.1 | -46.8 ± 13.1 | -47.3 ± 13.5 | 0.06 |  |
| **EQ5D** |  |  |  |  |  |
| **Preoperative** | 45.3 ± 13.7 | 44.3 ± 11.8 | 44.7 ± 12.6 | 0.13 |  |
| **Change at 3 m** | 26.5 ± 15.9 | 27.2 ± 12.3 | 26.9 ± 13.8 | 0.38 |  |
| **Change at 6 m** | 30.2 ± 16.2 | 30.9 ± 13.2 | 30.6 ± 14.5 | 0.40 |  |
| **Change at 1 year** | 31.7 ± 16.0 | 32.7 ± 13.9 | 32.3 ± 14.8 | 0.16 |  |

TKA, total knee arthroplasty; AKS, American Knee Society; WOMAC, Western Ontario and McMaster Universities Osteoarthritis Index; EQ5D, EuroQol 5-Dimension

Changes are calculated as the difference between postoperative scores and baseline (preoperative) values. WOMAC is reported in a decreasing direction (lower scores indicate better outcomes).
